# Sustained microparticle-mediated intracellular immunomodulation enhances tolerogenic dendritic cell stability and antigen-specific Treg induction

**DOI:** 10.3389/fimmu.2026.1897468

**Published:** 2026-09-14

**Authors:** Sihan Jia, Zachary Weiser, Meera Rajasekaran, Lia DiMitri, Peter Deak

**Affiliations:** 1 School of Biomedical Engineering, Drexel University, Philadelphia, PA, United States; 2 Department of Material Science and Engineering, Drexel University, Philadelphia, PA, United States; 3 Department of Chemical and Biological Engineering, Drexel University, Philadelphia, PA, United States

**Keywords:** cell therapy, dendritic cell, immune tolerance, microparticle, tolDC

## Abstract

Tolerogenic dendritic cells (tolDCs) are a promising cell therapy platform for restoring immune tolerance, but their clinical efficacy is limited by poor phenotypic stability and short *in vivo* persistence following transfer. Here, we developed biodegradable poly(lactic-co-glycolic acid) (PLGA) microparticles co-loaded with ovalbumin (OVA) and a combinatorial Push/Pull Immunomodulation (PPI) formulation consisting of the TLR7/8 agonist resiquimod (R848) and the JAK2 inhibitor CEP-33779. We hypothesized that sustained intracellular release of immunomodulators from phagocytosed microparticles would stabilize tolDC phenotype and enhance antigen-specific regulatory T cell (Treg) responses. PPI microparticles (PPI MPs) demonstrated sustained cargo release over 30 days, with the most pronounced pH sensitivity observed for CEP-33779, and were efficiently internalized by murine bone marrow-derived dendritic cells (BMDCs) and human monocyte-derived dendritic cells. BMDCs loaded with PPI MPs exhibited prolonged viability, increased PD-L1 and IL-10 expression, and resistance to inflammatory reprogramming following lipopolysaccharide challenge compared to dexamethasone-loaded microparticles or soluble drug controls. Administration of PPI MPs during BMDC differentiation further enhanced tolerogenic programming, producing marked increases in PD-L1 and IL-10 while suppressing TNFα secretion. Following adoptive transfer *in vivo*, PPI MP-treated DCs demonstrated prolonged persistence and accumulation in draining lymph nodes for up to 7 days. Finally, PPI MP-treated DCs induced robust antigen-specific Foxp3+ regulatory T cell responses in OVA-sensitized mice and suppressed antigen recall responses ex vivo. Together, these findings demonstrate that sustained intracellular combinatorial immunomodulation can generate a durable and functionally stable tolDC phenotype with enhanced antigen-specific tolerogenic activity, supporting this platform as a potential strategy for autoimmune therapies.

## 1 Introduction

Autoimmune diseases affect approximately 10% of the population in industrialized countries, and incidence has risen across most major categories over the past two decades (1, 2). Standard of care remains broad immunosuppression, which controls inflammation but elevates the risk of opportunistic infection, malignancy, and end-organ injury (3). Restoring tolerance to specific self-antigens, without compromising protective immunity, is therefore a central goal of current immunotherapy development (4–6).

Foxp3^+^ regulatory T cells (Tregs) are the principal mediators of peripheral tolerance, and antigen-specific Treg expansion is the central objective of these efforts (7–9). Among cell therapies pursued for this purpose, autologous tolerogenic dendritic cells (tolDCs) — an immunosuppresive DC substate typically characterized by low co-stimulation, high PD-L1, BTLA, IL-10 production and most critically Treg induction (10, 11) — are the most clinically advanced (12). TolDC cell therapies are typically generated by differentiating monocytes into immature DCs ex vivo, conditioning the cells with a single tolerogenic agent (typically dexamethasone, vitamin D3, rapamycin, or IL-10) (13), pulsing with autoantigen, and reinfusing them into the patient.

Phase I/II trials of this approach in type 1 diabetes (14), rheumatoid arthritis (15–17), Crohn’s disease (18), and multiple sclerosis (19) have demonstrated safety and antigen-specific immunomodulation but inconsistent and short-lived clinical responses (12). Current Phase IIa work is focused on administration route and dose (16). A central limitation is the phenotypic instability of conventional tolDCs after transfer: their tolerogenic state depends on continuous exposure to the conditioning agent, and recipient inflammatory signals — particularly TNF-α and TLR ligands — drive maturation and selectively deplete tolerogenic DC subsets (12, 20), narrowing the *in vivo* window for productive T-cell engagement. We hypothesize that improving this therapy would require both a more intrinsically stable tolerogenic program and a means to maintain that program *in vivo*.

We and others have shown that combinatorial activation of pattern-recognition, cytokine, and co-inhibitory pathways can produce a more stable tolerogenic phenotype than any single agent (21, 22). We previously reported Push/Pull Immunomodulation (PPI), in which DCs receive paired immune-agonist/immunomodulator combinations that increase inhibitory outputs (PD-L1, IL-10) while suppressing co-stimulatory and inflammatory outputs (CD80, CD40, TNFα); a liposomal PPI formulation reduces disease severity in murine experimental autoimmune encephalomyelitis (EAE) (22). A subsequent screen of >40,000 such combinations identified PPI-9 — the TLR7/8 agonist resiquimod (R848) paired with the JAK2 inhibitor CEP-33779 — as a lead (23, 24). In human CD14^+^ moDC assays, PPI-9 treated DCs adopt a transcriptional state distinct from dexamethasone- or LPS-conditioned controls and selectively expand antigen-specific Foxp3^+^ Tregs in autologous co-culture (25–27). In addition to this Treg formulation, we have also observed an improvement for both phenotypic stability and longevity *in vitro*, it is likely that tolDCs generated from soluble PPI will still not demonstrate sufficient persistence *in vivo*.

Encapsulating immunomodulators in biodegradable particles is one strategy to extend their intracellular activity (28, 29). PLGA microparticles are biocompatible, are efficiently internalized by professional antigen-presenting cells, and release encapsulated cargo over days to weeks via polymer hydrolysis (30, 31). Release kinetics are tuned by polymer molecular weight, lactide: glycolide ratio, and particle size (32), and accelerated under the acidic pH within endosomal compartments that follows phagocytosis (33). PLGA microparticles taken up by monocytes are retained intracellularly for several weeks and release sufficient drug to sustain an anti-inflammatory macrophage phenotype after adoptive transfer (31), a strategy recently extended to therapeutic control of macrophage polarization in pro-inflammatory and pro-fibrotic disease (34). PLGA particles co-loaded with autoantigen and a tolerogenic small molecule prevent or reverse disease in preclinical models of type 1 diabetes (35), EAE (36), and FVIII-inhibitor formation in hemophilia A (37, 38); co-encapsulation of antigen and tolerogenic signal in the same particle is required for stable, antigen-specific Treg induction (37). Building on these observations, we developed PPI MPs: PLGA microparticles co-loaded with PPI (R848 + CEP-33779) and ovalbumin (OVA) as a model antigen. DCs that phagocytose PPI MPs (DC^PPI MP^) release both cargos intracellularly over several weeks, sustaining a tolerogenic phenotype while ensuring that antigen and tolerogenic signal reach the same DC after transfer.

Here, we test whether DC^PPI MP^ functions as a more durable autologous tolDC therapy than DCs conditioned with soluble PPI or with dexamethasone-loaded PLGA microparticles (Dex MPs), the most clinically advanced single-agent comparator (39). We synthesized PPI MPs co-loaded with PPI and OVA and characterized particle size, morphology, loading, and pH-dependent release. We assessed PPI MP uptake by murine bone-marrow–derived DCs (BMDCs) (40) and human moDCs (27) during and after differentiation, and quantified surface PD-L1, CD80, IL-10, and TNFα in DC^PPI^ MP over 15 days, with or without LPS challenge to represent inflammatory cues after transfer. *In vivo*, we tracked DC^PPI^ MP residence in skin and migration to draining lymph nodes following subcutaneous injection in C57BL/6 mice (41), and tested whether DC^PPI^ MP induce antigen-specific Foxp3^+^ Tregs in OVA-sensitized recipients. Together, these experiments evaluate if intracellular sustained release of combinatorial immunomodulation can extend the functional lifetime of tolerogenic DC therapy.

## 2 Results

### 2.1 PPI MPs load PPIs, show sustained release and are internalized by DCs

We hypothesized that sustained intracellular release of PPI within a DC via PLGA MP delivery would increase the longevity and Treg generative capacity of a tolDC cell therapy (**Figure 1A**). Our first goal of this study was to synthesize and characterize PPI MPs (31). We selected Resomer 504H PLGA as the base polymer as it has been shown to have a steady degradation rate intracellularly. PLGA MPs of approximately 2 µm diameter were synthesized using single emulsion/sonication as previously described (29). PPI MPs were loaded with R848, CEP33779 and OVA whereas Blank MPs only contained OVA (**Table 1**). All MPs were loaded with 0.1% wt. DiD dye for tracking and quantification. All MPs were dialyzed for 24 h against PBS prior to use.

**Figure 1:**
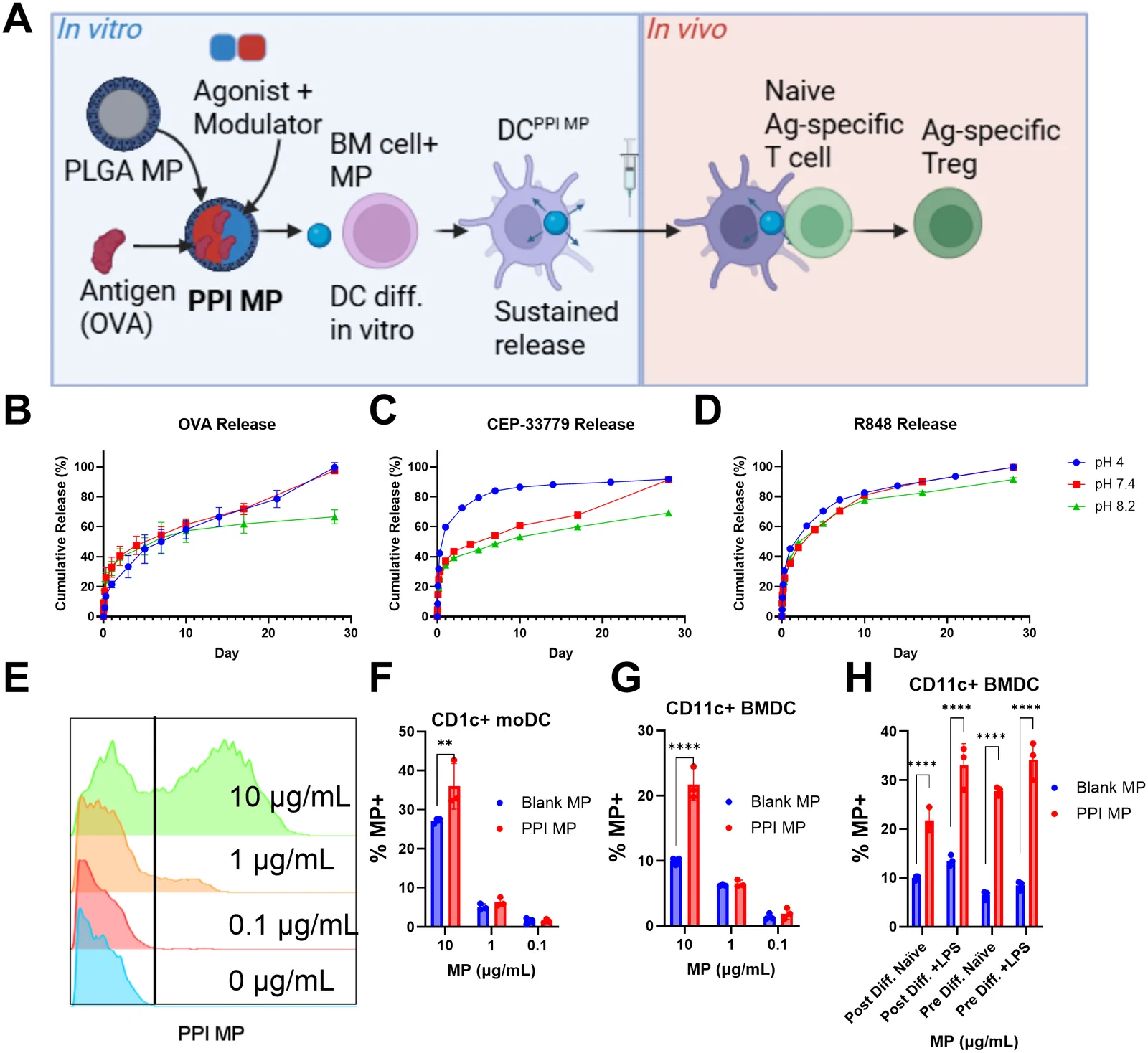
PPI MP Synthesis and Characterization. **(A)** Schematic image showing proposed mechanism of action for DC^PPI MP^. Left (blue box, *In vitro*): a PLGA microparticle is co-loaded with OVA antigen and the agonist-plus-modulator pair (blue and red blocks: the TLR7/8 agonist R848 and the JAK2 inhibitor CEP-33779) to form the PPI MP. Bone marrow cells internalize the PPI MP and are differentiated *in vitro* into dendritic cells (DC^PPI MP^) that release the cargo intracellularly in a sustained manner (arrows). Right (red box, *In vivo*): after subcutaneous injection, DC^PPI MP^ present antigen to naive antigen-specific T cells and drive their conversion into antigen-specific Tregs. **(B–D)** *In vitro* release of **(B)** OVA, **(C)** CEP-33779 and **(D)** R848 in Acetate buffer at pH 4 or pH 8.2 or PBS at pH 7.4 for PPI MP. **(E)** Representative histograms of differentiated CD1c+ human moDCs treated with DiD labeled PPI MPs for 24 h at various concentrations, washed and analyzed via flow cytometry. **(F)** MP+ populations of CD1c+ human moDCs from or **(G)** CD11c+ murine BMDCs after treatment for 24 h with PPI MP or Blank MPs. **(H)** Differentiated BMDCs (Post Diff. Naïve), BMDCs treated with LPS for 24 h (Post Diff. +LPS) were treated with 10 µg/mL of PPI MP or Blank MP for 24 h. Bone marrow cells (BMs) were also treated with 10 µg/mL of PPI MP or Blank MP during differentiation and analyzed (Pre Diff. Naïve) or treated with 100 ng/mL LPS for 24 h (Pre Diff. +LPS). All samples were washed and analyzed via flow cytometry. Error bars indicate ± SD of triplicate measurements. Significance was determined by two-way ANOVA with tukey test for multiple comparisons. **p<0.01, ****p<10^-4^.

**Table 1:** Description of PPI combination.

*Compound*	*Target*	*Type*	*IC_50_/EC_50_*
CEP-33779^23^	JAK2	Inhibitor	1.5 ng/mL
R848^24^	TLR7	Agonist	33 ng/mL
	TLR8	Agonist	100 ng/mL

MPs were analyzed for physical characteristics and molecule loading. Scanning electron microscopy (SEM) and dynamic light scattering (DLS) analysis confirmed an approximate 2 µm average diameter, with a slightly larger hydrodynamic diameter and moderate polydispersity, as some aggregation occurred in PPI MP samples (**Table 2**; **Supplementary Figures 1**, **2**). A negative zeta potential was observed from both blank and PPI MP, as expected (**Table 2**). Fourier transform infrared spectroscopy (FTIR) confirmed polymerization of PLGA but limited signals for PPI drugs or OVA, indicating low surface presentation (**Supplementary Figures 3A–C**). This is further supported by electron dispersive spectroscopy (EDS) analysis, which confirmed majority oxygen and carbon on MP surfaces with >1% sulfur but no nitrogen, indicating only a small presence of OVA protein for both Blank and PPI MP (**Supplementary Tables 1**, **2**). Further testing confirmed colloidal stability approximately 4 h and limited dissolution in PBS over 9 days for both MP formulations, consistent with other PLGA MP studies (**Supplementary Figures 3D, E**) (42). HPLC analysis of MP extracts showed a 1:4 drug loading ratio of CEP-33779 to R848 and a loading of approximately 3.4% by weight of OVA (**Table 3**). All three cargos exhibited sustained release over 30 days (**Figures 1B–D**). CEP-33779 showed the most pronounced pH dependence, with faster release at pH 4 than at pH 7.4 or 8.2 (**Figure 1C**). OVA and R848 showed smaller differences among the tested pH conditions and did not exhibit the same marked acid-accelerated release profile (**Figures 1B, D**), indicating cargo-dependent release behavior. (**Figures 1B–D**; **Supplementary Figure 4**) (43).

**Table 2:** MP sizing by SEM and DLS.

*Sample*	*Diameter (µm, SEM)*	*PDI*	*Diameter (µm, DLS)*	*PDI*	*Zeta Potential (mV)*
PPI MPs	2.05 ± 1.61	0.62 ± 0.02	2.65 ± 0.12	0.11 ± 0.01	−29.4 ± 10.1
Blank MPs	1.58 ± 1.02	0.42 ± 0.04	2.09± 0.07	0.10 ± 0.01	−19.2 ± 6.6

**Table 3:** PPI MP loading data.

*Compound*	*PPI MP*		*Blank MP*	
	*Encapsulation Efficiency (%)*	*Loading (wt. %)*	*Encapsulation Efficiency (%)*	*Loading (wt. %)*
CEP-33779	18.02 ± 0.79	1.01 ± 0.04	N/A	N/A
R848	1.79 ± 0.09	4.03 ± 0.02	N/A	N/A
OVA	13.8 ± 2.0	3.4 ± 0.48	18.0 ± 2.0	3.2 ± 0.36

We observed internalization of MPs by both murine bone marrow derived DCs (BMDCs) and human monocyte derived DCs (moDC). MoDCs or BMDCs at 100k per well were incubated with either Blank MPs (OVA loaded only) or PPI MPs, demonstrating increased uptake at 10 µg/mL for PPI MPs (**Figures 1E–G**). We further evaluated the effect of adding MPs during differentiation or after and the effect of LPS maturation on MP uptake. Murine BMDCs were treated with 10 µg/mL PPI or Blank MPs for 5 days during GM-CSF differentiation or 24 h after differentiation both with and without an additional 24 h treatment with 100 ng/mL lipopolysaccharide (LPS) to mature BMDCs. Cells were then washed and analyzed via flow cytometry showing increased uptake of PPI MPs compared to Blank MPs and showing higher uptake of cells treated pre-differentiation (**Figure 1H**).

### 2.2 PPI MPs improve tolerogenic markers and longevity *in vitro* in differentiated BMDCs

After validating that PPI MPs are internalized by DCs *in vitro*, we analyzed the effect of PPI MP on long term viability and phenotypic stability on differentiated DCs. BMDCs were generated as previously published by isolating bone marrow cells from C57Bl/6 mice and then culturing for 5 days *in vitro* with GM-CSF. On day 6, BMDCs were incubated with varying concentrations of PPI MPs, Blank MPs or MPs loaded with dexamethasone (Dex MP). Dexamethasone (dex) is the most common immunomodulatory deployed in tolDC differentiation and is a critical control both within MPs and as a free drug (39, 44, 45). We confirmed dex loading into PLGA MPs along with OVA (**Supplementary Table 3**). We also included free drug controls for Dex and PPI equivalent to the total drug amount in 10 µg/mL MP treatments. 24 h after MP treatment, DCs were washed once to remove excess MPs and then incubated for 1, 3, 5, 7, 10, 12 or 15 days *in vitro*. During incubation, media was refreshed every 2–3 days with fresh GM-CSF. At given timepoints, cells were analyzed for viability using XTT assay, analyzed by flow cytometry for DC markers CD11c/MHCII, the co-stimulatory marker CD80 and the tolerance marker PD-L1. Additional groups were challenged with 100 ng/mL LPS for 24 h and then analyzed via flow cytometry and ELISA for tolerogenic cytokine IL-10 and inflammatory cytokine TNFα (**Figure 2A**).

**Figure 2:**
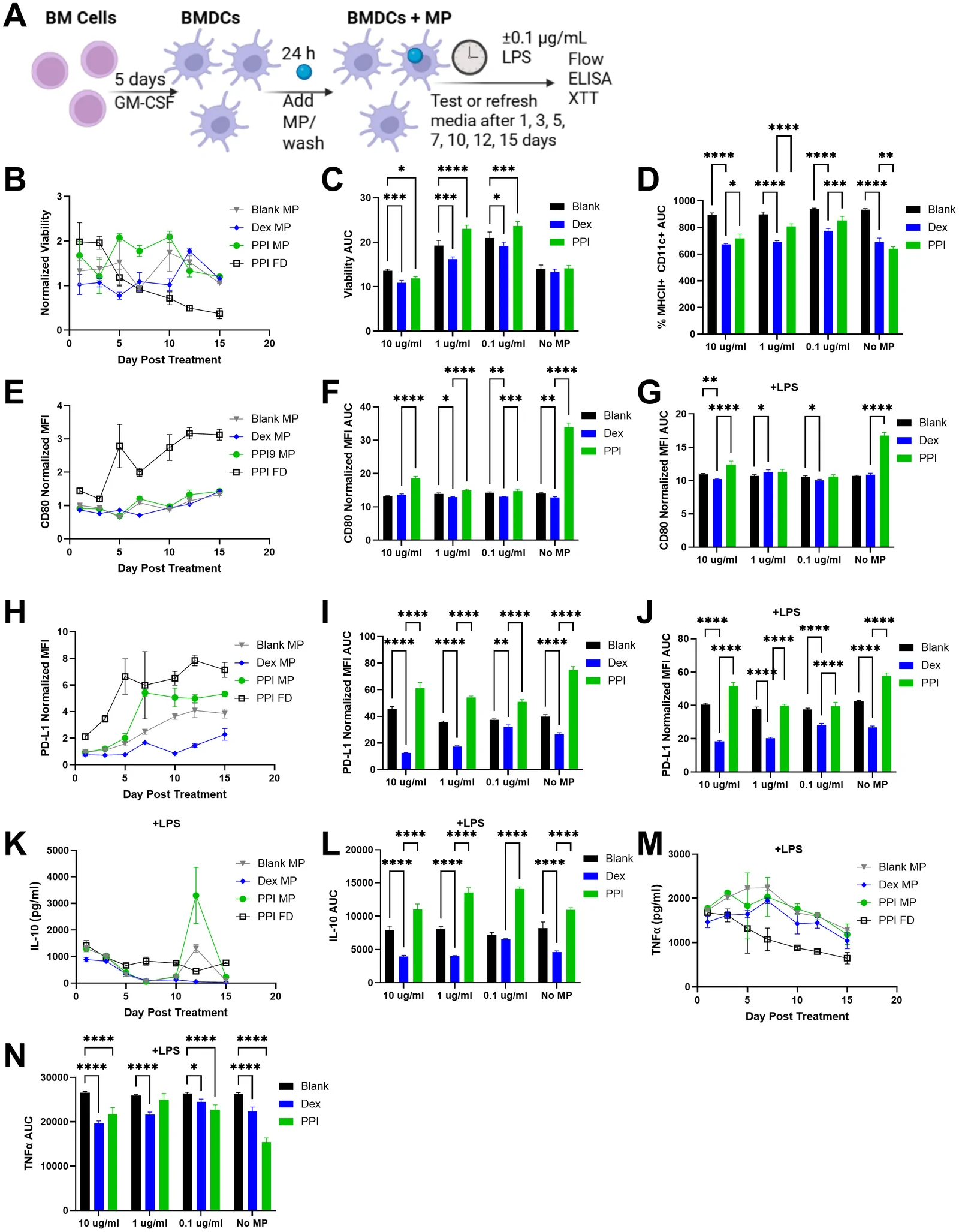
PPI MPs improve longevity and tolerogenic markers of differentiated BMDCs *in vitro*. **(A)** Experimental schematic. Freshly isolated bone marrow (BM) was isolated from C57Bl/6 mice and then differentiated into BMDCs after 5-day culture in GM-CSF. BMDCs were then incubated with Blank MP, Dex MP or PPI MPs at 10, 1 or 0.1 µg/mL or free drug (FD) equivalent to 10 µg/mL condition as controls (No MP) for 24 h. Cells were then washed and incubated for 1, 3, 5, 7, 10, 12 or 15 days with media refreshed at each timepoint. Some groups were additionally treated with 100 ng/mL LPS for 24 h prior to testing. **(B)** Viability normalized to untreated controls over time as determined by XTT assay. **(C)** Area under the curve (AUC) of data from part **(B, D)** % MHCII+, CD11c+ AUC for 15 days. **(E)** CD80 mean fluorescence intensity (MFI), normalized to untreated controls on day 1 post treatment over time. **(F)** AUC for data from **(E, G)** AUC of CD80 MFI after LPS stimulation normalized to BMDCs treated only with LPS. **(H)** PD-L1 normalized MFI over time. **(I)** AUC of normalized PD-L1 MFI. **(J)** AUC of PD-L1 MFI for LPS treatment groups. **(K)** IL-10 concentration in culture supernatants collected at each indicated time point after a 24h challenge with 100 ng/mL LPS. Each point represents the mean of three replicate cultures from a single time-course experiment; error bars indicate SD. **(L)** Area under the curve of the 15-day IL-10 time course shown in **(K, M)** TNFα in supernatants on indicated day after LPS treatment. **(N)** AUC for data in part **(M)** Error bars indicate ± SD of triplicate measurements. Significance was determined by two-way ANOVA with tukey test for multiple comparisons. *p<0.05, **p<0.01, ***p<10^-3^ ****p<10^-4^.

The results of this experiment showed that overall PPI MP improved DC longevity and tolerogenic markers *in vitro*. We observed increased viability, especially prior to day 10 for BMDCs treated with 1 µg/mL PPI MPs, compared to Dex MP or Blank MP, which we quantified by calculating the area under the curve (AUC) of the viability over the 15 day experiment (**Figures 2B, C**). Over the 15-day time course, PPI MP-treated BMDCs retained a higher AUC for the percentage of CD11c^+^MHCII^+^ cells than Dex MP- or soluble PPI-treated cells, whereas Blank MP-treated cells showed the highest values. Because this endpoint reflects the frequency of the phenotypically defined CD11c^+^MHCII^+^ population, it should not be interpreted as a direct measure of tolerogenic function.

(**Figure 2D**; see **Supplementary Figure 5** for gating strategy) (46). The co-stimulatory marker, CD80, was only slightly upregulated for PPI MP over the study, whereas free drug PPI generated a nearly 3-fold increase after 5 days both with and without LPS stimulation, suggesting MP release restrains immunological reprogramming (**Figures 2E–G**). The opposite effect was observed for PD-L1, increasing to a 5-fold increase over untreated controls on day 7 and remaining elevated regardless of LPS stimulation (**Figures 2H–J**). The effect on PD-L1 also increased with increasing PPI MP concentration. After LPS stimulation, we measured both IL-10 and TNFα release. IL-10 output varied across the 15-day time course and showed a transient elevation at day 12 before declining by day 15 (**Figure 2K**). This peak at day 12 was rather surprising and should be interpreted with caution. Further supporting this as a real effect, however, we observe elevated IL-10 for PPI MPs for most timepoints and a similar day 12 IL-10 rebound was observed at 10 µg/mL PPI MP (**Figures 2K, L**; **Supplementary Figure 6**). The TNFα levels were mostly unchanged by MP treatment, although 10 and 0.1 µg/mL PPI MP or Dex MP treatment observed a modest decrease in TNFα release compared to Blank MP controls (**Figures 2M, N**). **Supplementary Figure 6** contains additional data used to generate AUC curves for all treatment groups.

Overall, this data demonstrated that PPI MPs can increase the functional tolerance markers PD-L1 and IL-10 with having a minimal effect on CD80 and TNFα, even in response to inflammatory LPS challenge. PPI MPs also increased longevity and phenotypic stability. It is notable, but not unexpected, that the Blank MP formulations also increased PD-L1 and IL-10, as it is known that PLGA MPs are tolerogenic on their own, although this effect was cooperative with PPI (47).

### 2.3 PPI MPs further increase PD-L1 and IL-10 responses in BMDCs if administered during differentiation

While PPI MPs induce PD-L1 and IL-10 expression when given to naïve BMDCs, we hypothesized that PPI MPs may generate a more profound tolDC phenotype if administered during differentiation process. Many protocols administer tolerizing molecules during differentiation process to further bias tolerogenic transcriptional programming when cells are in a more stem-like state as a bone marrow precursor cell (48). Furthermore, bone marrow precursor phagocytosis, while relatively understudied, has been known to generate more tolerant downstream immune phenotypes (49, 50). To test this hypothesis, we administered PPI MP or controls during GM-CSF differentiation process immediately after bone marrow isolation (Pre-Diff) or after GM-CSF differentiation as in **Figure 2**. We then either tested using similar analyses as in **Figure 2** immediately after differentiation or 5 days later to understand how this phenotype alters with time.

We observed that viability was still relatively stable on day 5 whether treated Pre or Post-Diff. For 10 µg/mL PPI MP and soluble PPI, the viability was significantly higher when the treatment was delivered during differentiation (Pre-Diff.) than when it was delivered after differentiation (Post-Diff.) (**Figure 3A**). While we tested 10, 1 and 0.1 µg/mL PPI, Dex and Blank MPs both Pre/Post-Diff at both timepoints (**Supplementary Figure 7**), the most interesting results compared Pre-Diff to Post-Diff for the 10 µg/mL PPI MP groups. Pre-Diff. PPI MP treatment produced an approximately two-fold increase in CD80 MFI immediately after differentiation. After five additional days of culture (Pre-Diff.+D5), CD80 declined and was no longer significantly different from the corresponding untreated control (**Figure 3B**). PD-L1, however, increased dramatically when PPI was administered prior to differentiation either in MPs or free drug. There was a >10-fold increase in PD-L1 for PPI MP on day 1 that increased to >15 fold on day 5 (**Figure 3C**). In contrast to **Figure 2** which showed a modest impact on TNFα, PPI MP and PPI FD significantly reduced LPS mediated-TNFα secretion on both day 1 and day 5 (**Figure 3D**). Similar improvement was also observed in IL-10 secretion; PPI MPs and PPI FD improved IL-10 secretion on day 1 but only PPI MPs retained IL-10 expression into day 5. Directly comparing Pre/Post-Diff. for PPI MPs, there was a highly significant increase in tolerogenic markers PD-L1 and IL-10 with minimal change in inflammatory markers either at day 1 or 5 (**Figures 3F, G**). Taken together, we concluded that treating BMDCs with PPI MPs during differentiation process would improve the stability and magnitude of tolerogenic markers. Finally, to further evaluate this as a translational strategy, we validated that PPI MP treated DCs can undergo a freeze/thaw cycle and retain PD-L1 expression and viability (**Supplementary Figure 8**).

**Figure 3:**
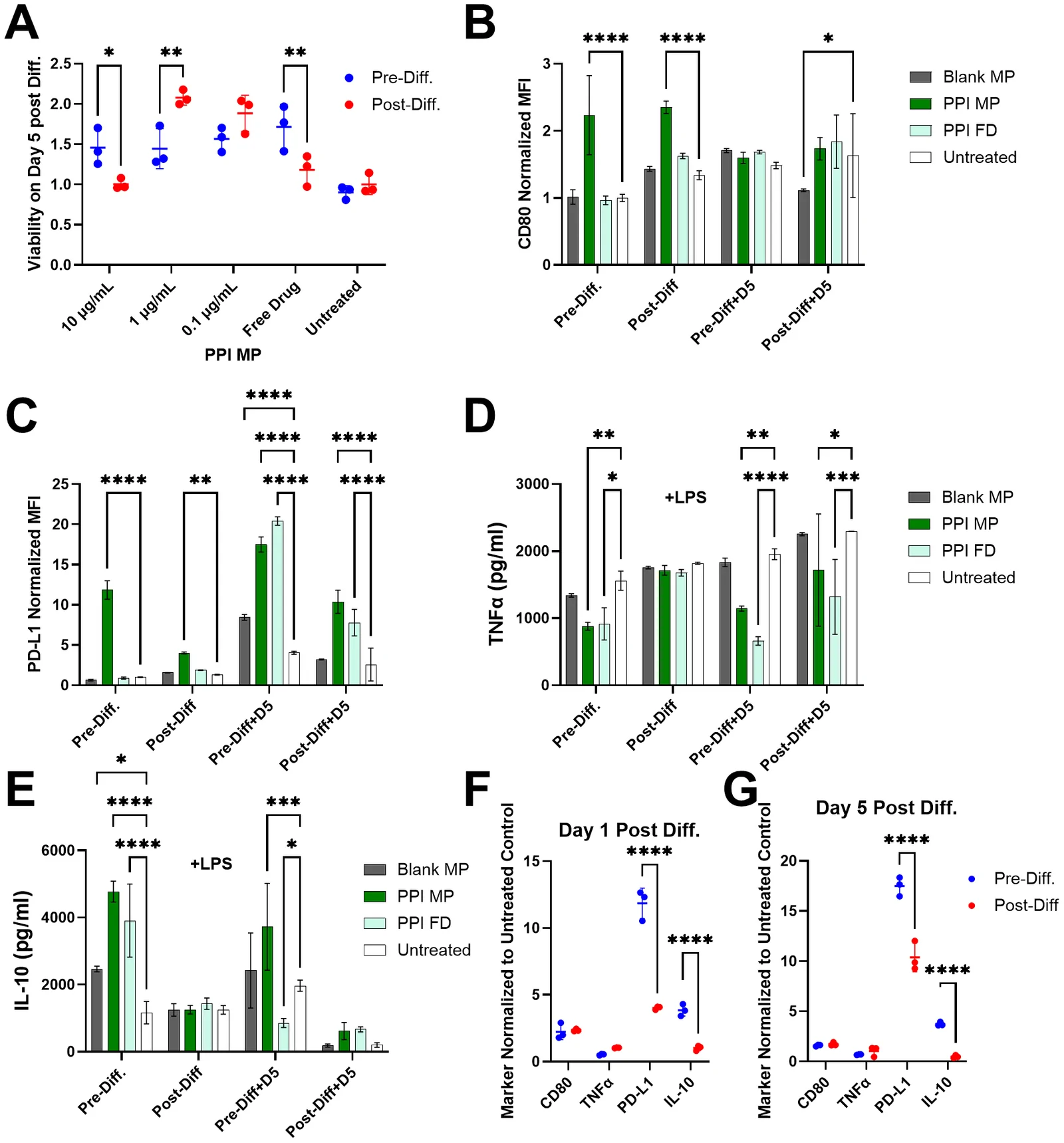
PPI MPs upregulate tolerance markers more effectively when administered during BMDC differentiation *in vitro*. Blank MP or PPI MP (10, 1, 0.1 µg/mL) or free drug equivalent were administered either during differentiation on D0 (Pre-Diff) or after differentiation as in **Figure 2** (Post-Diff). Cells were then either washed tested 24 h later or incubated for 5 additional days in fresh cell culture media and tested (+D5). **(A)** Viability on day 5 via XTT assay. Significance bars in panel A denote the Pre-Diff. versus Post-Diff. comparison within each treatment condition. **(B)** CD80 MFI values were normalized to the mean of the Pre-Diff. untreated group., **(C)** PD-L1 MFI. **(D)** TNFα or **(E)** IL-10 after 24 h incubation with LPS. For B-E, all groups were treated with 10 µg/mL only. See **Supplementary Figure 6** for full dataset. **(F)** Day 1 or **(G)** Day 5 direct comparisons of Pre-Diff and Post-Diff DCs treated with 10 µg/mL PPI MP. Error bars indicate ± SD of triplicate measurements. Significance was determined by two-way ANOVA with tukey test for multiple comparisons. *p<0.05, **p<0.01, ***p<10^-3^ ****p<10^-4^.

### 2.4 DC^PPI MP^ induce OT-II specific Tregs and suppress OT-I proliferation

As a final *in vitro* validation, we demonstrated that PPI MP treated DCs can generate Tregs and suppress T cell proliferation *in vitro*. Because MPs were loaded with OVA, we chose to test MP loaded DCs with OVA-specific transgenic OT-I (C57BL/6-Tg(TcraTcrb)1100Mjb/J) and OT-II (B6.Cg-Tg(TcraTcrb)425Cbn/J) mice, which only express T cell receptors specific for the major MHCI OVA epitope (OT-I) or MHCII OVA epitope (OT-II) and are commonly used to test antigen specificity (51, 52).DCs were similarly generated from C57Bl/6 mice as in **Figure 3**, pre-treated with 1 µg/mL OVA for 30 minutes and then co-cultured at 1:5 (DC:T cell) with T cells magnetically isolated from OT-I or OT-II mouse spleens. Cells were incubated for 6 days and then analyzed for OT-II Treg differentiation and OT-I T cell proliferation. We observed a significant increase of OT-II specific Tregs compared to naïve DCs, higher than any other treatment group (**Supplementary Figures 9A, B**). We also observed a significant decrease in OT-I T cell proliferation compared to naïve DCs, like free dex and PPI treated DCs (**Supplementary Figures 9C, D**). We also observed decreases in CD69+ T cells, a marker of early T cell activation (53). These results indicate that DCs treated with MPs suppress T cell proliferation and induce Tregs in an antigen specific fashion.

### 2.5 DC^PPI MP^ traffic to lymph nodes

After evaluating the phenotype of DCs pretreated with PPI MPs, now referred to as DC^PPI MP^, *in vitro*, we next performed an *in vivo* experiment both to track DC^PPI MP^ migration to draining lymph nodes (LN), which is a critical step for the differentiation of naïve T cells into antigen specific Tregs, and track *in vivo* DC^PPI MP^ viability (54). Murine BM cells were isolated and then incubated with DiD labeled 10 µg/mL of PPI MPs, Blank MPs or Dex MPs or free drug equivalent of R848/CEP-33779 plus GM-CSF for differentiation (PPI FD). BMDCs were differentiated for 5 days, washed, stained with a DiO lipophilic tracking dye and then injected s.c. into C57Bl/6 mice in each flank (0.5 x 10^6^ DCs per injection; **Figure 4A**). At 1, 3, 5, and 7 days post injection, mice were sacrificed and skin near injection site and draining inguinal LN disaggregated and analyzed by flow cytometry gating on live, CD11c+ cells for LN and all live cells for skin (**Supplementary Figures 10A, B**). It is known that membrane labels, such as DiO, and MPs can be internalized by endogenous APCs via active membrane transfer or after cell death (55, 56). Labeling of both DC membranes and MPs allowed for tracking of MP+ and MP- DC as well as independent tracking of MP, increasing confidence that the injected cells are being directly tracked.

**Figure 4:**
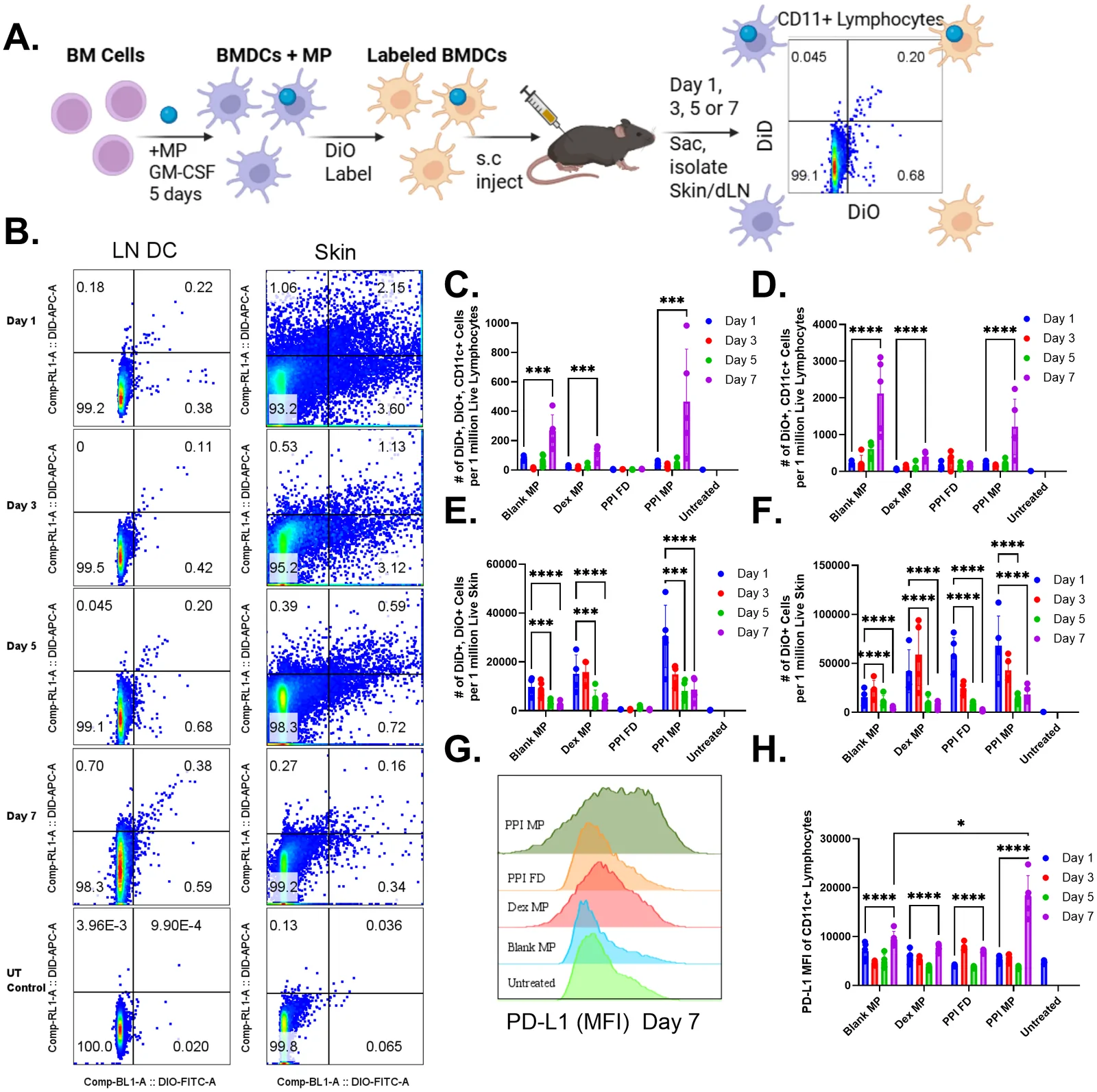
PPI MP loaded DCs drain to lymph nodes *in vivo* after adoptive transfer. **(A)** Experimental Schematic. Bone marrow (BM) cells were treated with DiD labeled blank MP, Dex MPs, PPI MPs at 10 µg/mL or free drug equivalent for PPI FD and differentiated with GM-CSF for 5 days. Cells were then stained with DiO and injected s.c. in both flanks at 5x10^5^ cells per injection (1x10^6^ cells per mouse). Mice were sacrificed 1, 3, 5, and 7 days after injection and skin near injection site and inguinal lymph nodes isolated, disaggregated and analyzed by flow cytometry. **(B)** Representative flow plots on day 1, 3, 5 and 7 for PPI MP DC groups gated on live, CD11c+, lymphocytes (left) and live skin cells (right) for DiD (MP+) and DiO (injected cells). Untreated control mouse is included at bottom. **(C)** Number of DiD+, DiO+, CD11c+ lymphocytes or **(D)** Number of DiO+, CD11c+ lymphocytes per 10^6^ live lymphocytes. **(E)** Number of DiD+, DiO+ or **(F)** DiO+ live skin cells per 10^6^ live skin cells. **(G)** Representative flow plot of **(H)** PD-L1 MFI for CD11c+ lymphocytes from treatment groups on day 7. N = 4–5 mice per group, error bars indicate ± SD, significance was determined by two-way ANOVA with tukey test for multiple comparisons. *p<0.05, ***p<1x10^-3^, ****p<10^-4^.

Our results indicate that MP laden DCs migrate more than expected. We observed that all injected DC (DiO+) and MP+ DC (DiD+, DiO+) populations generally decreased in skin and increased in LN over time for both DC^Bl MP^ and DC^PPI MP^, peaking on day 7, whereas DCs treated with free PPI or Dex MP treated DCs demonstrated limited drainage to LN (**Figures 4B–F**; **Supplementary Figures 10C–J**). The general trends for both MP+ DCs and total injected DC were consistent in DC^PPI MP^, suggesting that PPI MPs had limited impairment of LN drainage (**Figures 4C, D**). The total number of DiD+, DiO+ DCs that migrated to LNs were highest for DC^PPI MP^ groups, suggesting that PPI MP treatment improved migration, although DC^Bl MP^ also showed significant LN drainage on day 7. An analysis of all CD11c+ lymphocytes revealed a significant increase in PD-L1 for all groups on day 7, which was highest for the DC^PPI MP^ (**Figures 4G, H**; **Supplementary Figure 10K**). There was also an increase in overall CD11c+ cells in LN on day 7 for DC^PPI MP^ (**Supplementary Figure 10L**). This result suggests that DC^PPI MP^ both migrate and retain their tolerizing potential even after 7 days *in vivo*. It should be noted that only 20-50% of MP+ cells were CD11c+, despite injected cells being >80% CD11c+ (**Supplementary Figure 8M**). While this effect is potentially explained by incomplete MP uptake by DCs, direct MP transfer between cells, downregulation of CD11c or internalization of apoptotic DCs, MP+ cells are still overrepresented in the CD11c+ compartment (2-8% of lymphocytes), indicating that injected cells do migrate to LNs (57). We further confirmed that DC^PPI MP^ had increased CCR7 expression compared to DC^Dex MP^ prior to injection, a critical LN migration marker, suggesting that this increase migration was due to CCR7-CCL21 chemotaxis (**Supplementary Figure 10N**) (58). This is not surprising, as it has been shown previously that MP internalization can promote CCR7 expression (50). Taken together, this data indicates that DC^PPI MP^ have improved *in vivo* viability and LN migration compared to Blank MP, Dex MP or FD PPI treated DCs.

### 2.6 DC^PPI MP^ generates antigen-specific Tregs

After confirming that DC^PPI MP^ migrate to LNs, we designed an experiment to observe how DC^PPI MP^ impacts T cell responses, hypothesizing that DC^PPI MP^ would increase OVA specific Tregs. C57Bl/6 mice (N = 5/group) were vaccinated i.p. with 10 µg R848/100 µg OVA on day 1 and then boosted on day 14 to generate OVA specific immunity. Mice were then injected 0.5x 10^6^ DCs into each flank s.c. as in **Figure 4** with DC^PPI MP^ or controls on day 17 and 21, then sacrificed on day 30 and draining inguinal LNs isolated disaggregated and analyzed via flow cytometry (**Figure 5A**). Groups included: 1) OVA-only control, mice treated with 100 µg/mouse as untreated control, 2) DC^Bl MP^ to control for effect of MP 3) DC^Dex^, to benchmark PPI against standard immunomodulator, 4)Free PPI drug equivalent (PPI FD) to control for free drug effects, 5) DCs treated with PPI FD (DC^PPI FD^) to control for sustained PPI release, 6) our experimental group, DC^PPI MP^, or 7) untreated, naïve C57Bl/6 mice as a negative control (**Figure 5B**). Using flow cytometry, we identified Tregs (Live, Single, CD45+, CD3+, CD4+, CD25+, CD127^lo^, FoxP3+), CD8+ T cells, DCs (CD11c+) and the major MHCI (OVA_257-264_) and MHCII (OVA_323-339_) tetramer positive T cell populations (**Supplementary Figure 11**). We first noted that DC^Dex^ had a significant decrease in overall Treg populations whereas DC^PPI MP^ increased Tregs (**Figure 5C**). Looking at OVA specific Tregs, this effect was more pronounced, with a nearly 2-fold increase of OVA_323–339_ tetramer+ Tregs per total lymphocytes over all controls in DC^PPI MP^ groups (**Figure 5D**). DC^PPI MP^ treatment did not alter OVA_257–264_ tetramer+ CD8+ T cells or overall OVA_323–339_ tetramer+ CD4+ T cells compared to other DC groups (**Figures 5E, F**). We calculated an over 1:1 ratio of OVA specific Tregs to all OVA specific CD4+ T cells and a >3:1 ratio of OVA specific Tregs to OVA specific effector CD8+ T cells (**Figures 5G, H**).

**Figure 5:**
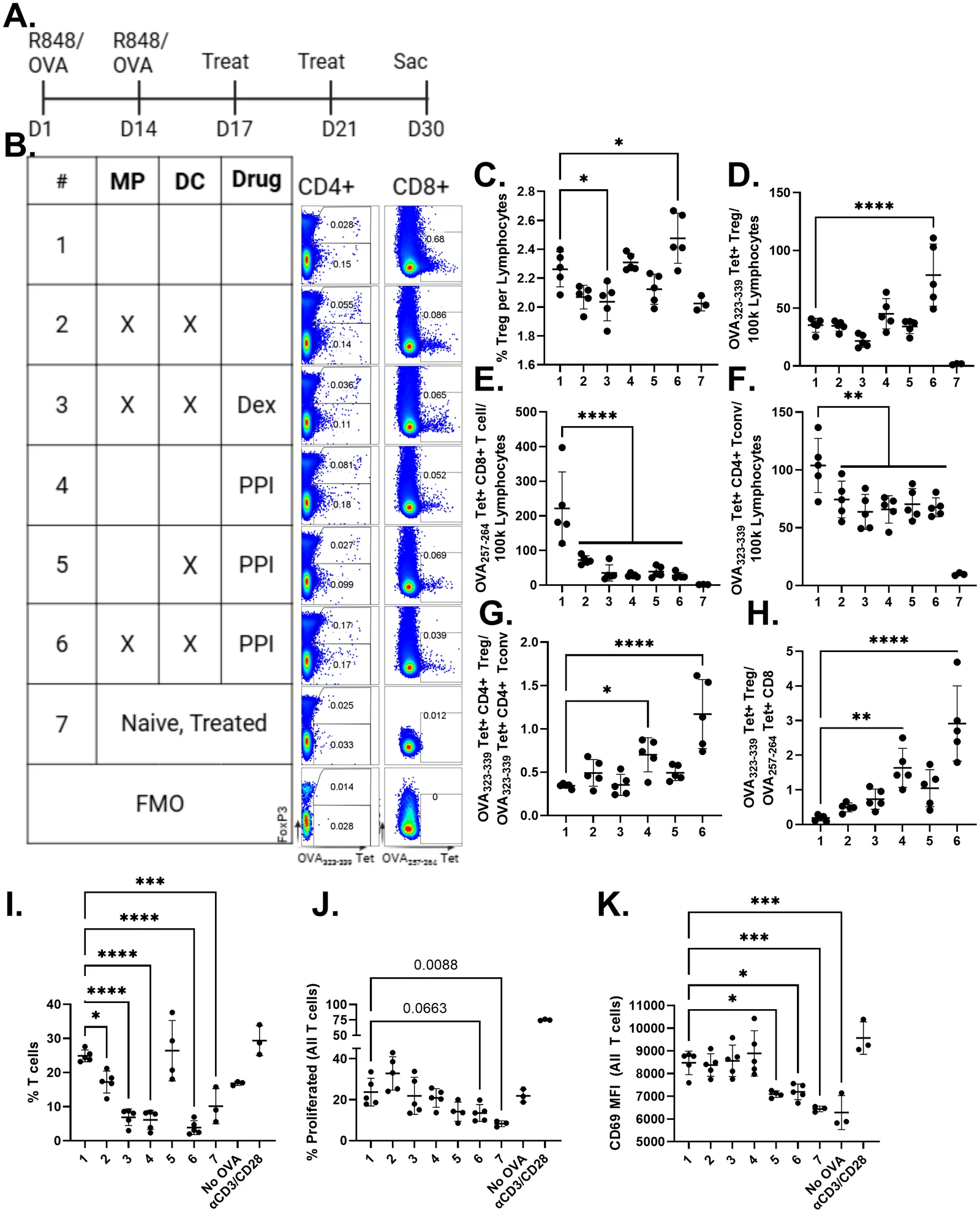
DC^PPI MP^ generates active OVA specific Treg responses *in vivo*. **(A)** Experimental timeline of OVA/R848 sensitization and treatment. C57Bl/6 mice were sensitized on day 1 and day 14 with 10/100 µg/mouse of R848/OVA injected i.p. Mice were then treated with DC formulations or controls on day 17 and 21, then sacrificed on day 30 and draining LN harvested. All mice were injected with total 1x10^6^ DCs (0.5x10^6^ per flank) or injected with equivalent FD PPI or 100 µg OVA i.p. **(B)** Table indicating treatment groups if they contain MP, DC and any drugs (left) representative flow plots showing gating of OVA_323–339_ MHCII tetramer (x-axis) by FoxP3 (y-axis) of CD4+ T cells (middle) and gating of OVA_257–264_ MHCI tetramer (x-axis) by side scatter (SSC, y-axis) of CD8+ T cells (right). **(C)** % Total Treg (CD3+, CD4+, CD25+, CD127^lo^, FoxP3+) cells of live singlet, CD45+ lymphocytes. **(D)** Number of OVA_323–339_ MHCII tetramer Tregs per100k live, singlet CD45+ lymphocytes **(E)** Number of OVA_257–264_ MHCI tetramer CD8+ T cells per100k live, singlet CD45+ lymphocytes. **(F)** Number of OVA_323–339_ MHCII tetramer CD4+ Tconv (CD45+, CD3+, CD4+, FoxP3-) per100k live, singlet CD45+ lymphocytes. **(G)** Ratio of tetramer+ Tregs/Tconv. **(H)** Ratio of tetramer+ Tregs/CD8+ T cells. **(I)** Lymphocytes were then stained with cell proliferation stain and stimulated ex vivo (100k per well) with 1 µg/mL OVA for 6 days. Pooled lymphocytes from all samples not stimulated with OVA and stimulated with 1 µg/mL anti-CD3/CD28 antibody were used as negative and positive controls. % T cell (CD45+, CD3+, Live Singlets) **(J)** % proliferation CD3+ cells. **(K)** CD69 MFI of all CD45+, CD3+ T cells on day 6. N = 3–5 mice per group, error bars indicate ± SD, significance was determined by two-way ANOVA with tukey test for multiple comparisons. *p<0.05, ***p<1x10^-3^, ****p<10^-4^.

We further analyzed markers of Treg effector function, PD-1 and CTLA-4 (59). Representative histograms from the DC^PPI MP^ group showed right-shifted PD-1 and CTLA-4 distributions in OVA323–339 tetramer^+^ Tregs relative to bulk T cells (**Supplementary Figures 12A, B**). The percentage of PD-1^+^ cells within the total live CD45^+^CD3^+^ T-cell compartment was increased in the DC^PPI MP^ group (**Supplementary Figure 12C**), whereas no corresponding significant difference was observed in CTLA-4^+^ frequency (**Supplementary Figure 12D**). This analysis shows that the PD-1 difference was detectable in the total T-cell compartment but does not distinguish antigen-specific from antigen-nonspecific T cells. When gated on Tregs, we observed an expected increase in PD-1 compared to all T cells in all groups but only observed increases in CTLA-4 for Tregs from DC^PPI MP^ groups (**Supplementary Figures 12E, F**). This suggests that DC^PPI MP^ can not only increase Tregs and antigen-specific Tregs but also improve functional markers. We hypothesize that this increased tolerizing effect is due to improved DC^PPI MP^ migration as we also observed increased total number of DCs in draining LNs compared to all controls, whereas total B cell populations were unchanged (**Supplementary Figures 12G, H**).

To further evaluate the suppressive capacity of DC^PPI MP^, Cell Proliferation Dye eFluor 670-stained (Thermo) lymphocytes were cultured for 6 days and stimulated by 1 μg/mL OVA. Cells were analyzed for T-cell composition, proliferation, and CD69 expression. **Supplementary Figure 13A** shows the gating strategy; panels B/C show the CD4^+^/CD8^+^ composition of the CD3^+^ compartment; panels D/E show proliferation in the CD4^+^ and CD8^+^ subsets; and panels F/G show CD69 MFI in the corresponding subsets. The subset-level proliferation and CD69 analyses complement the total T-cell readouts shown in **Figure 5J** and **Figure 5K**, respectively (53). We observed a significant decrease compared to OVA treated group in total T cell populations, T cell proliferation and CD69 MFI for DC^PPI MP^ groups, suggesting limited antigen recall response (**Figures 5I–K**). The T cell proliferation responses of DC^PPI MP^ was even similar to naive lymphocytes proliferation after OVA challenge. These results indicate that DC^PPI MP^ drive functional and antigen suppressing Tregs.

## 3 Discussion

Here we presented a novel strategy for induced tolDC with high phenotypic resilience and longevity *in vivo* by MP-mediated sustained intracellular release of PPI. We have shown that PPI MPs effectively load and release drug and are internalized by DCs *in vitro* (**Figures 1B–H**). Using extensive *in vitro* analysis over the course of 15 days, we demonstrated that PPI MP treated BMDCs improve *in vitro* viability and retain tolerogenic markers IL-10 and PD-L1, while not increasing inflammatory markers TNFα and CD80 (**Figures 2B–N**; **Supplementary Figure 6**). This effect was pronounced by adding PPI MP prior to BMDC differentiation (**Figures 3B–G**; **Supplementary Figure 6**). When injected *in vivo*, DC^PPI MP^ migrated to LN over 7 days (**Figures 4B–H**; **Supplementary Figures 10C–L**) and generated antigen-specific Treg responses with increased PD-1 (**Figures 5C–H**; **Supplementary Figures 12A–F**). Taken together, this data supports DC^PPI MP^ as a promising translational therapy for antigen-specific immune tolerance (**Figures 5C–K**; **Supplementary Figure 12**, **13**).

While generation of translational immunosuppressive therapies was our explicit goal at study outset, DC^PPI MP^ leverages three distinct tolerizing mechanisms that seem to function cooperatively: 1) a unique drug combination (PPI), 2) sustained release of MPs and 3) tolerizing effect of PLGA MPs. This design has generated some interesting results we will discuss further. It is known that PLGA MPs modulate APCs in absence of any immunogenic signal. Getts et al. showed that antigen loaded PLGA MPs promote Treg tolerance in absence of immunomodulator (47). It is also known that PLGA MPs can specifically promote tolerance via sustained antigen exposure (60). This cooperative effect is immunologically important but has yet to translate into a functional therapy. We hypothesize that this effect, while measurable, does not persist within inflammatory contexts such as autoimmunity or transplantation rejection, which can rapidly reprogram tolDCs (20). We have shown that PPI treated DCs are more resilient to inflammatory reprogramming and generating functional tolerance (25, 26). In study, we show that this effect is improved by loading PPI into PLGA MPs (**Figures 2B–N**, **3B–G**).

Another noteworthy aspect of PPI-induced tolDCs is a lack of CD80 suppression (**Figures 2E–G**, **3B, F, G**). Although CD80 is traditionally viewed as a co-stimulatory molecule, CD80 also binds CTLA-4 with high affinity and contributes to Treg-mediated suppressive function, suggesting that moderate CD80 expression may be compatible with, or even supportive of, tolerogenic DC activity (61). Our results contribute to a growing body of literature suggesting that moderate or mild co-stimulatory signals on tolDCs are essential for Treg generation (62, 63). We further hypothesize that this balance between activating and regulatory signaling achieved from PPI is the reason DC^PPI MP^ retain their tolerogenic phenotype even after immunogenic LPS challenge (**Figures 2G, J-N**, **3D–G**).

*In vivo*, DC^PPI MP^ also had interesting effects. We initially hypothesized that DC^PPI MP^ treatment would primarily enhance local persistence near the injection site relative to other tolDC formulations, rather than promote sustained LN trafficking beyond several days. The prolonged migration of DC^PPI MP^ to draining lymph nodes over a 7-day period was unexpected (**Figures 4B–F**; **Supplementary Figures 10C–J**). Conventional DC migration to draining lymph nodes typically occurs within 1–2 days following activation; however, prior studies have shown that tolerogenic DCs exposed to immunomodulatory agents frequently exhibit impaired lymph node homing due to reduced CCR7 expression and responsiveness (64–66). Additionally, MP internalization has shown conflicting effects previously. DCs containing micron-scale particles have been reported to exhibit reduced migratory capacity (67). Conversely some studies indicate that phagocytosis of MPs can induce CCR7 expression and activation (50). Here, we observed DC^PPI MP^ both increased CCR7 and showed delayed migration over 7 days (**Figures 4B–F**; **Supplementary Figures 10C–J, L**).

An alternative explanation for the observed LN signal is that endogenous APCs internalized apoptotic debris derived from transferred cells and subsequently trafficked to the lymph node (68). Although this possibility cannot be excluded based on the presented data, several observations argue against a purely artifact-driven explanation. Specifically, the presence of a distinct double-positive (DiO+, DiD+) population suggests co-localization of both the membrane label and microparticles within the same cells rather than nonspecific background signal (**Figures 4C–F**; **Supplementary Figures 10C–J**). Furthermore, if LN accumulation were solely attributable to apoptotic debris uptake, similar signals would be expected in the DC^Dex MP^ and DC^PPI FD^ groups, which were not observed (**Figures 4B–F**; **Supplementary Figures 10C–J**). Finally, DC^PPI MP^ exhibit many hallmarks of LN homing, such as CCR7 (58), but also moderate levels of CD80 and high PD-L1 (**Figures 2E–J**, **3B, C, F, G**, **4G, H**; **Supplementary Figures 10K, L**). It is less well-known that CD80 and PD-L1 can undergo cis-interactions on DC cell surfaces and this PD-L1/CD80 pair contributes to LN homing (69).

Nevertheless, we acknowledge that fluorescent tracking alone cannot definitively distinguish intact donor-cell migration from secondary cargo transfer. Future studies incorporating genetic lineage-tracing approaches will be necessary to more rigorously define the fate and migratory behavior of transferred cells and track drainage for longer timepoints (70). Despite these limitations, the present findings demonstrate an unexpected degree of lymph node trafficking in the DC^PPI MP^ group that warrants further mechanistic investigation (**Figures 4B–H**; **Supplementary Figures 10C–L**) and conclude that this LN trafficking is a contributing factor for the increased antigen-specific Treg responses in LNs (**Figures 5C–H**; **Supplementary Figures 12G, H**).

This study also provided other opportunities for future work. While we demonstrate robust antigen-specific Treg (**Figures 5C–H**), we do not demonstrate that DC^PPI MP^ treats or prevents any disease model, instead focusing on the DC phenotypic analysis for this study. This lays the foundation for future studies to measure DC^PPI MP^ efficacy in autoimmune or transplantation disease models. Furthermore, this study utilized mostly murine BMDCs; future work should test more translatable human models, such as moDCs (25). Finally, there is a significant opportunity to further evaluate the effects of MP uptake on bone marrow cells and more rigorously analyze how MP uptake alters bone marrow cell differentiation even in the absence of immunomodulatory agents.

## 4 Materials and methods

### 4.1 Materials

All fluorescently tagged antibodies, αCD28/CD3 antibodies, ELISA kits, RBC Lysis Buffer, Foxp3/Transcription Factor Staining Buffer Set, EDTA, Cell Proliferation Dye eFluor™ 670, DiO, DiD cell labeling dye were purchased from Thermo Scientific. All chemicals for MP synthesis were purchased from Sigma unless otherwise noted. R848, CEP-33779, dexamethasone (>95% pure) were purchased from MedChemExpress. Bovine Serum Albumin (BSA) was purchased from VWR Life Science. HBSS, DPBS, PBS, DMEM, RPMI 1640, AIM-V medium, FBS, HIFBS, HEPES, Non-Essential Amino Acids Solution were purchased from Gibco. EndoFit Ovalbumin (OVA) was purchased from InvivoGen. Spleen Dissociation Medium was purchased from StemCell. β-mercaptoethanol was purchased from MP Biomedicals. Cell Activation Cocktail (without Brefeldin A), Recombinant Mouse GM-CSF (carrier-free)(20 ng/ml) and human IL-4 and GM-CSF and all plates unless noted otherwise were purchased from Thermo Fisher Scientific. H2-Kb-SIINFEKL-APC (MHCI OVA tetramer) and I-Ab, QAVHAAHAEIN-PE (MHCII OVA tetramer) were obtained from the NIH Tetramer Facility.

### 4.2 Methods

#### 4.2.1 MP synthesis

PPI, and blank microparticles (only OVA-loaded) were synthesized via a single emulsion strategy (29). In short, 20mg of PLGA was solved in 1mL of a 9:1 dichloromethane (DCM): trifluoroethanol (TFE) solution in a glass vial. 2 µL of DiD (1,1’-dioctadecyl-3,3,3’,3’- tetramethylindocarbocyanine perchlorate), a fluorescent lipophilic dye, 20 mg of R848 and 0.5 mg of CEP-33779 (only for PPI MP) were added or 25 mg of Dex added. The organic solution was sonicated lightly for 30 seconds on ice, then 4mL of a 2% PVA aqueous solution containing 3.3 mg OVA was added to the organic phase. A probe sonicator (Polytron PT 3100 D) set at 10,000 rpm emulsified the solution, forming oil-in-water droplets. After sonication, an additional 5mL of the 2% PVA solution was added to mixture. The solution was placed uncovered under a fume hood on a stir plate and set to stir at 1,200 rpm for 6 hours, then washed with DI water, aliquoted, flash frozen, freeze dried and UV sterilized until use.

#### 4.2.2 Physical analysis of MPs

##### 4.2.2.1 Dynamic light scattering and zeta potential analysis

Particle size, polydispersity index (PDI), and zeta potential of PLGA MPs were characterized using a Zetasizer Nano ZS (Malvern Panalytical, Malvern, UK). For particle size analysis, PLGA MPs were suspended at 100 μg/mL in 1 mL of 1× phosphate-buffered saline (PBS), gently mixed to obtain a homogeneous suspension, and transferred to disposable polystyrene cuvettes. Hydrodynamic diameter and PDI were measured by dynamic light scattering using a 633 nm He-Ne laser with 173° backscatter detection at 25 °C. The Z-average hydrodynamic diameter and PDI were calculated using cumulant analysis.

For zeta potential measurements, PLGA MPs were suspended at 100 μg/mL in 1 mL of 1 mM KCl and loaded into disposable folded capillary zeta cells (DTS1070, Malvern Panalytical). Electrophoretic mobility was measured by laser Doppler electrophoresis using M3-PALS technology, and zeta potential was calculated using the Smoluchowski model. All measurements were performed at 25 °C in triplicate, and data acquisition and analysis were conducted using ZEN software (Malvern Panalytical). Results are reported as the mean ± standard deviation.

##### 4.2.2.2 Scanning electron microscopy and electron dispersive spectroscopy analysis

Particle size was determined by SEM and atomic surface composition by EDS using a Thermo Fisher Apreo 2S Lo Vac SEM. Samples were dissolved at 0.5 mg/mL in DI water and dried on SEM stub then sputter-coated with a platinum (80%) and palladium (20%) using a Cressington 208HR Sputter Coater to enhance image contrast. Size and PDI were analyzed using Fiji Image J software analyzing >1000 particles at either 1000x or 2000x magnification.

##### 4.2.2.3 Fourier transform infrared spectroscopy

The chemical composition of PLGA MPs was characterized by Fourier transform infrared (FTIR) spectroscopy using a Thermo Nicolet Nexus 870 FT-IR spectrometer (Thermo Fisher Scientific) equipped with a single-bounce attenuated total reflectance (ATR). Dry PLGA MP samples or raw components were placed directly onto the ATR crystal, and spectra were collected over the 4000–650 cm^-^¹ spectral range at 4 cm^-^¹ resolution, with 32 scans averaged per sample. Background spectra were collected prior to each measurement and automatically subtracted. Spectra were processed using the instrument software and analyzed to confirm the characteristic functional groups of PLGA, including ester carbonyl (C=O), C–O–C, and aliphatic C–H stretching vibrations.

#### 4.2.3 Colloidal stability and dissolution kinetic analysis of MPs

Colloidal Stability. Colloidal stability of PLGA microparticles (MPs) was evaluated by monitoring particle sedimentation over time. MPs were suspended in 1× phosphate-buffered saline (PBS) at a concentration of 1 mg/mL and gently vortexed to obtain a homogeneous suspension. Immediately following dispersion, the suspension was transferred to a NanoDrop One UV-Vis Spectrophotometer (Thermo Fisher Scientific), and the absorbance at 650 nm was measured at predetermined time points as particles settled. Changes in absorbance were used as an indirect measure of suspension stability, with decreasing absorbance indicating particle sedimentation.

Dissolution Kinetics. The dissolution behavior of PLGA MPs was assessed by suspending particles in 1× PBS and incubating them under physiological conditions. At designated time points over a 9-day period, particle hydrodynamic diameter and polydispersity index (PDI) were measured by dynamic light scattering (DLS) using a Zetasizer Nano ZS (Malvern Panalytical, Malvern, UK) as described above. Changes in particle size over time were used to monitor particle degradation and dissolution kinetics. All measurements were performed in triplicate, and data are reported as the mean ± standard deviation.

#### 4.2.4 Drug loading analysis

For calculation of drug loading, 1 mg of MP was dissolved in 2 mL of DCM and sonicated for 30 minutes, rotovapped to remove DCM, then extracted with 50/50 ACN/water and analyzed via Agilent 1260 Infinity II HPLC system with an analytical C8 column and a gradient of 5-95% ACN in 20 minutes, comparing to free drug controls. For OVA loading, 1 mg of MP was dispersed in 500 µL of DCM and 500 µL of PBS + 0.1% tween 20 and sonicated for 5 minutes three times, DCM removed and analyzed with BCA protein quantification kit (Thermo Scientific).

#### 4.2.5 Monocyte derived dendritic cell differentiation

MoDCs were differentiated according to prior literature (27). Human monocytes were sourced from the Human Immunology Core facility (RRID: SCR_022380) at the University of Pennsylvania. All samples were obtained from anonymized donors, following strict compliance with informed consent guidelines and prior approval from the Institutional Review Board (IRB) at the University of Pennsylvania (IRB# 832307). Human monocytes were cultured in RPMI 1640 supplemented with 10% heat-inactivated FBS and 1× Penicillin-Streptomycin. Cells were seeded at 1 million per ml and differentiated with 100 ng/mL GM-CSF and 100 ng/mL IL-4 at 37 °C and 5% CO_2_. On day 3, half of fresh culture medium containing GM-CSF and IL-4 was added. Differentiated moDCs were used for downstream experiments on day 5. Matured moDCs were treated with 100 ng/mL LPS for 24 h.

#### 4.2.6 Use of animals

6-week C57BL/6 female mice were housed and treated according to approved IACUC protocol (No. LA-25-056) by Drexel University Laboratory Animal Resources.

#### 4.2.7 BMDC differentiation

BMDCs were generated according to previous literature (26). Freshly isolated murine bone marrow (BM) cells were cultured in complete medium - RPMI 1640, 10% HI-FBS, recombinant mouse GM-CSF (carrier-free, 20 ng/mL; BioLegend), 2 mM L-glutamine, 1% antibiotic-antimycotic, and 50 µM β-mercaptoethanol - and differentiated for 5 days prior to use. For experiments in **Figures 4** and **5**, all BMDCs were co-treated with MPs or controls during 5 day differentiation process.

#### 4.2.8 BMDC *in vitro* analysis

MP uptake and the *in vitro* tolerogenic phenotype were evaluated in BMDCs. For uptake, BMDCs (1 × 10^5^ cells/well) were incubated with DiD-labeled blank, PPI, or Dex MPs at 10, 1, or 0.1 µg/mL for 24 h, washed to remove non-internalized particles, and the DiD^+^ (MP^+^) fraction of CD11c^+^ cells quantified by flow cytometry. To test how the timing of MP delivery and the maturation state of the DC affect uptake and phenotype, MPs were added at four points relative to differentiation, each analyzed 5 days after MP addition: at the start of differentiation (day 0; Pre-Diff.), mid-differentiation (day 3), immediately after differentiation (Post-Diff.), and to Pre and post-differentiation DCs maintained an additional 5 day period in culture before treatment (Pre-Diff+D5 and Post-Diff+D5). Half of the medium was replaced with fresh GM-CSF–supplemented medium every 3 days (BMDC culture as in 4.2.7). All conditions were run in triplicate with blank, PPI, or Dex MP at 10, 1, and 0.1 µg/mL, alongside PPI and Dex free-drug equivalent of 10 µg/mL and untreated cells as controls, and each was analyzed with and without a 24 h challenge with 100 ng/mL LPS to model an inflammatory environment. The tolerogenic phenotype was assessed by staining CD11c^+^ DCs for CD80, PD-L1, BTLA, and MHC II; culture supernatants were collected for IL-10 and TNF-α ELISA; and cell viability was measured in parallel by XTT assay.

##### 4.2.8.1 Flow cytometry (*in vitro*)

Cells were dispensed into 96-well V-bottom plates, blocked with anti-mouse CD16/CD32 (20 min, 4 °C), and stained with the surface antibody panel (1 h, 4 °C). Antibodies were diluted in staining buffer (HBSS, 1% BSA, 0.1 mM EDTA). Cells were washed twice, resuspended in fresh staining buffer, and acquired on an Attune NxT cytometer.

##### 4.2.8.2 Cytokine ELISA

Secreted IL-10 and TNF-α in culture supernatants were quantified with commercial sandwich ELISA kits (Thermo Fisher Scientific) according to the manufacturer’s protocol, and optical density was read at 450 nm with a 620 nm reference on a microplate reader.

##### 4.2.8.3 Cell viability (XTT)

Metabolic viability was assessed by XTT assay: XTT labeling reagent was added to cultures and incubated at 37 °C for 4 h, and formazan absorbance was read at 450 nm with a 620 nm reference. Viability was normalized to untreated control cells.

#### 4.2.9 DC *in vitro* longevity analysis

To evaluate the durability of the tolerogenic phenotype, BMDCs were treated after differentiation with blank, PPI, or Dex MP at 10, 1, or 0.1 µg/mL (or the free-drug equivalent of 10 µg/mL) and maintained in culture, replacing half of the medium with fresh GM-CSF - supplemented medium every 3 days (BMDC culture as in 4.2.7). At 1, 3, 5, 7, 10, 12, and 15 days after treatment, cells were left naïve or challenged with 100 ng/mL LPS for 24 h and analyzed for CD11c, CD80, PD-L1, BTLA, and MHC II as in 4.2.8. Cells were plated in 96-well flat-bottom plates at 2 × 10^5^ per well (200 µL) for flow phenotyping and at 1 × 10^5^ per well (200 µL) for XTT viability. At each timepoint, supernatants were assayed for IL-10 and TNF-α by ELISA and viability by XTT, as described in 4.2.8.

#### 4.2.10 OT-I and OT-II *in vitro* assays

Splenocytes from OT-I (C57BL/6-Tg(TcraTcrb)1100Mjb/J) or OT-II (B6.Cg-Tg(TcraTcrb)425Cbn/J) mice were isolated using 1 mg/mL collagenase D with 0.1 mg/mL DNAse I in RPMI media for 30 minutes similar to previously published procedures (26). Murine T cells were isolated for the single cell splenocyte mixture using a negative T cell selection kit (StemCell Technologies, Catalog # 19851) according to manufacturer’s instructions. DCs were treated with MPs or free drugs during differentiation process as previously described in **Figure 3**, plated at 20,000 cells per well in T cell media (RPMI, 1x Glutamax, 10% HIFBS, 1x NEAA, 50 µM Beta-Mercaptoethanol, 1 mM EDTA, 1x Pen-Strep), incubated for 30 minutes with 1 µg/mL OVA and then co-cultured with 100,000 total OT-I or OT-II T cells. OT-I cells were stained with cell proliferation eflour670 (Thermo) prior to co-culture. Cells were incubated for 72 h, ½ of media exchanged on day 3 and then analyzed on day 6 post co-culture. Cells were then stained for Treg and T cell proliferation as previously published (26).

#### 4.2.11 *In vivo* DC migration analysis

Mice were injected with labeled BMDCs for the experiment in **Figure 4** to track *in vivo* longevity and LN drainage. BMDCs were harvested as before, differentiated/treated, then on day 5, washed and stained with 1 µg/mL DiO cell staining dye (Thermo) for 15 minutes, washed with cold HBSS 1x and then injected into each side of a female C57Bl/6 mouse flank s.c., 0.5x10 (6) total cell per injection, 10^7^ cells/mL into a roughly 3 x 3 cm pre-shaved area of skin. 1, 3, 5, and 7 days later, mice were sacrificed and both inguinal LNs and a 3x3 cm area of skin centered around injection site cut into 0.5 x 0.5-inch pieces harvested. Single cell suspensions were prepared by incubation with Spleen Dissociation Medium (StemCell) for 30 min for lymphocytes and 2 h for skin at 37 °C and 5% CO2, then passed through a 70 μm filter, centrifuged at 200 g for 5 min and resuspended splenocytes in HBSS + 2% HIFBS + 1 mM EDTA. Lymphocytes were stained for CD11c and PD-L1. Both skin and lymphocytes were stained for viability using Live Dead Near IR Fixable Stain (Thermo) according to manufacturer protocol.

#### 4.2.12 Analysis of *in vivo* T cell responses

##### 4.2.12.1 Immunization

Female C57BL/6 mice (N = 5 per group) were sensitized intraperitoneally (i.p.) on days 1 and 14 with 10 µg R848 and 100 µg OVA per mouse to establish OVA-specific immunity.

##### 4.2.12.2 BMDC preparation and MP loading for therapy

BM cells were seeded at 5 × 10^6^ cells per dish in 10 mL complete medium supplemented with GM-CSF (20 ng/mL; complete medium as in 4.2.7). The following day, DiD/OVA-loaded blank MP, Dex MP, or PPI MP (retrieved from −80 °C and resuspended in PBS to 1 mg/mL, a 100× stock), or the free PPI drug equivalent, were added at 10 µg/mL (100 µL of 1 mg/mL stock per dish) and co-cultured with the BMDCs for 5 days prior to injection. Half of the medium was replaced with fresh GM-CSF-supplemented medium on day 2.

##### 4.2.12.3 Adoptive transfer and treatment

On days 17 and 21, mice were anesthetized with isoflurane (3% induction; 1.5-3% maintenance) and treated. For cell-therapy groups, BMDCs were harvested, washed with Ca^2+^/Mg^2+^-free HBSS, centrifuged (300 × g, 5 min), counted, assessed for viability, and resuspended at 1 × 10^7^ cells/mL. Cells were injected subcutaneously (s.c.) into both lateral caudal hind flanks using a 27 G insulin syringe at a shallow (~10–20°) angle to raise a localized subcutaneous bleb draining to the ipsilateral superficial inguinal LN (50 µL per flank; 0.5 × 10^6^ cells per flank; 1 × 10^6^ cells per mouse). Treatment groups were: (1) untreated naïve mice; (2) OVA only (100 µg OVA in 100 µL per mouse, i.p.); (3) free PPI drug equivalent (PPI FD; R848/CEP-33779 at the free-drug-equivalent dose, i.p.); (4) PPI MP alone, without cells (100ug/100ul/mouse PPI MP only, s.c.); (5) blank-MP BMDCs (DC^Bl MP^, s.c.); (6) Dex-MP BMDCs (DC^Dex MP^, s.c.); (7) PPI free-drug-treated BMDCs (DC^PPI FD^, s.c.); and (8) PPI-MP BMDCs (DC^PPI MP^, s.c.). All antigen-containing formulations included OVA. Mice were sacrificed on day 30.

##### 4.2.12.4 Tissue harvest and single-cell preparation

Draining inguinal LNs were harvested into RPMI. LNs were digested in RPMI containing 1 mg/mL Collagenase D and 0.1 mg/mL DNase I (37 °C, 20 min) and gently mashed with a syringe plunger. Spleens collected from OVA-only mice were mashed and digested in the same buffer for 40 min for use as single-color compensation controls. Digestion was stopped with 10 mM EDTA, suspensions were filtered through 70 µm strainers, and cells were washed in RPMI/staining buffer and DPBS. Splenocytes were centrifuged (200 × g, 7 min) and RBC-lysed (1× lysis buffer, RT, 10 min). LN cells were resuspended at 1 × 10^7^ cells/mL, and 2 × 10^6^ cells in 200 µL were plated per well in 96-well V-bottom plates (half of the LN cells were reserved for proliferation analysis, 4.2.12).

##### 4.2.12.5 Flow cytometry staining

Cells were stained with Live/Dead Fixable Near-IR (1:1000 in HBSS; 30 min, 4 °C), washed, Fc-blocked (FcX), and stained with MHC I (H2-Kb/OVA257-264, APC) and MHC II (I-Ab/OVA323-339, PE) tetramers (30 min, 4 °C). Cells were then surface-stained with the antibody cocktail in the presence of Fc block (1 h, 4 °C; **Supplementary Table 4**), fixed and permeabilized with the Foxp3/Transcription Factor Staining Buffer Set, and stained intracellularly for FoxP3 (overnight, 4 °C). Single-color and FMO compensation controls were prepared from spleen cells in parallel. Samples were washed and acquired on a BD FACSymphony A1. Tregs were defined as live, single, CD45^+^CD3^+^CD4^+^CD25^+^CD127lo FoxP3^+^ cells; CD8^+^ T cells, CD4^+^ conventional T cells (FoxP3^-^), CD11c^+^ DCs, and CD19^+^ B cells were also enumerated, along with the MHC I- and MHC II-tetramer^+^ populations and the functional markers PD-1 and CTLA-4.

#### 4.2.13 Ex vivo T cell proliferation analysis

Half of the LN cells harvested as in 4.2.11 were used for proliferation analysis and labeled with Cell Proliferation Dye eFluor™ 670 on the day of harvest. Cells were washed twice with HBSS to remove residual serum, resuspended at 2 × 10^6^ cells/mL in HBSS, and labeled by adding an equal volume of 10 µM dye (in room-temperature HBSS) while gently vortexing (5 µM final). Cells were incubated for 10 min at 37 °C in the dark, quenched with 4–5 volumes of cold complete medium (10% serum), held on ice for 5 min, washed twice in complete medium, and resuspended in T-complete medium consisting of RPMI 1640 supplemented with 10% HI-FBS, 1× Penicillin-Streptomycin, 1 mM HEPES, 1× nonessential amino acids, and 50 *μ*M *β*-mercaptoethanol. Dye-labeled LN cells (1 × 10^5^ per well) were cultured in U-bottom plates with 1 µg/mL OVA. Pooled lymphocytes from all samples, either left unstimulated or stimulated with 1 µg/mL anti-CD3/anti-CD28, served as negative and positive controls, respectively. After 3 days, half of the medium was replaced with fresh medium only, and on day 6 cells were stained with Live/Dead Fixable Near-IR followed by surface antibodies (**Supplementary Table 5**) and analyzed by flow cytometry.

#### 4.2.14 Statistical analysis

Statistical significance determined via using GraphPad Prism (version 10.0). All experiments were carried out in biological triplicates and p values <0.05 were determined to be significant and p values are listed on figures. P values were determined by two-way ANOVA with tukey *post hoc* test or two tailed student’s T test for single comparisons, with test used listed in the figure legend.

## Data Availability

The raw data supporting the conclusions of this article will be made available by the authors, without undue reservation.
