# Supplementary material for: Sustained microparticle-mediated intracellular immunomodulation enhances tolerogenic dendritic cell stability and antigen-specific Treg induction
Source: Front Immunol. 2026 Sep 14;17:1897468. doi: 10.3389/fimmu.2026.1897468 (PMC13616712; doi:10.3389/fimmu.2026.1897468)
Supplement: Supplementary file 1 [file Supplementaryfile1.docx]

Supplementary Material

# Sustained Microparticle-Mediated Intracellular Immunomodulation Enhances Tolerogenic Dendritic Cell Stability and Antigen-Specific Treg Induction

Supplementary Figures 1-13

Supplementary Table 1-5

**Supplementary Figures**

**Supplementary Figure 1.** Representative SEM images of PPI and Blank MPs.

**Supplementary Figure 2.** DLS analysis of (A) Blank MP and (B) PPI MP. Overlay of three technical replicates.

**Supplementary Figure 3.** Additional MP physical characterization. (A-C) Fourier transform infrared spectroscopy (FTIR) analysis of MP formulations (A) Blank MP, (B) Dex MP and (C) PPI MP. We observed characteristic PLGA polymer peaks at approximately 1100 cm^-1^ and 1750 cm^-1^. For Dex MP, we observed characteristic aromatic carbon peaks at 1650 cm^-1^ for Dex and N-H and O-H bond peaks at 3300-3400 cm^-1^ for OVA protein, suggesting surface presentation. These peaks were less pronounced for Blank and PPI MP, suggesting limited surface presentation of OVA and PPI. (D) Colloidal stability test. 1 mg/ml MPs were dispersed in PBS and absorbance at 650 nm monitored. All MPs fall out of solution in about 4 h. (E) Dissolution Kinetic Assay. MPs were incubated at 23 °C in PBS at 1 mg/mL for up to 9 days and average diameter tested (N=3 technical replicates) by DLS. Diameter did not significantly change over 9 days, suggesting limited dissolution in PBS.

**Supplementary Figure 4.** HPLC analysis of PPI MP extract showing elution of R848 and CEP-33779 with absorbance at 220 nm.

**Supplementary Figure 5.** Flow gating strategy for BMDCs analyzed in figure 2 and 3.

**Supplementary Figure 6.** Additional data from figure 2 showing all tested groups. (A) XTT viability assay. (B) % of MHCII, CD11c+ cells from single live cells, (C) CD80 MFI normalized to day 1 untreated controls, (D) same as C but with addition of LPS 24 h prior to testing. (E) similar to C, but stained for PD-L1, (F) plus LPS, (G) IL-10 or (H) TNFα concentrations in supernatants on indicated day after addition of LPS 24 h prior. Significance was determined by two-way ANOVA with tukey test for multiple comparisons. FD= free equivalent drug to 10 µg/mL MP dose. ****p<10^-4^.

**Supplementary Figure 7.** Additional data from figure 3 showing all tested groups. (A) XTT viability assay treated to MPs on day 1 of BMDC differentiation (Pre-Diff) or on day 5 after differentiation (Post-Diff). All cells were then let to rest in BMDC media for 5 days, then tested with XTT. (B) % of MHCII, CD11c+ cells from single live cells treated with MPs on day 1, 3 or 5 during differentiation, then tested on day 6, 24 h after finished differentiation. (C) CD80 normalized MFI, (D) PD-L1 normalized MFI, (E) TNFα or (F) IL-10 from groups treated Pre-Diff. or Post-Diff. then analyzed 24 h after differentiation or analyzed 5 days after differentiation completion. Significance was determined by two-way ANOVA with tukey test for multiple comparisons. FD= free equivalent drug to 10 µg/mL MP dose. ****p<10^-4^.

**Supplementary Figure 8**. Analysis of DC^PPI MP^ freeze thaw stability. MP treated DCs were generated as in figure 3. 1 million cells were frozen in 7.5% DMSO+ BMDC cell culture media for 4 days then rapidly thawed. Cells were incubated for 24 h in cell culture media with or without 100 ng/ml LPS and then analyzed by flow. (A) Viability as determined by trypan blue staining of frozen versus unfrozen cells. (B) % recovery of cells post freeze/thaw cycle. (C) PD-L1 MFI of frozen cells ±LPS. N=3 biological replicates, significance determined by one-way ANOVA. ****p<10^-4^.

Supplementary Figure 9. PPI MP treated DCs generate OT-II Tregs and suppress OT-I T cell proliferation in vitro. BMDCs were differentiated with 10 µg/mL blank, dex or PPI loaded MPs (Blank MP-DC, Dex MP-DC and PPI MP-DC) or free drug equivalent (naïve DC, Dex-DC, PPI FD-DC) for 5 days in vitro similar to Figure 3. Mature DC (mDCs) were generated by treating naïve DCs on day 5 with 100 ng/mL LPS for 24 h. After differentiation, cells were then washed, plated and treated with 100 µg/mL OVA protein for 30 minutes, then co-cultured 1:5 with T cells isolated from spleens of OT-I or OT-II mice. After 6 days of co-culture, T cells were analyzed for Treg differentiation and T cell proliferation. (A) Representative flow plots of CD25 vs. FoxP3 staining of live, CD3+, CD4+ OT-II splenocytes for PPI MP-DC, Dex-DC and No DC (T cell only) group. (B) Quantification of Treg populations. (C) Representative histograms of T cell proliferation stain of live, CD3+, CD8+ OT-I splenocytes for mature DCs, Dex-DC and PPI MP-DC treated groups. Line indicates cut-off between proliferated and non-proliferated. (D) Quantification of % proliferated. (E) % CD69+ of live, CD3+, CD8+ OT-I splenocytes. N=3 biological replicates, significance determined by one-way ANOVA. *p<0.05, **p<0.01, ***p<10^-3^, ****p<10^-4^.

**Supplementary Figure 10.** Supplemental data for in vivo DC adoptive transfer experiment. Bone marrow (BM) cells were treated with DiD-labeled blank MP, Dex MPs, PPI MPs at 10 µg/mL or free drug equivalent for PPI FD and differentiated with GM-CSF for 5 days. Cells were then stained with DiO and injected s.c. in both flanks at 5x10^5^ cells per injection (1x10^6^ cells per mouse). Mice were sacrificed 1, 3, 5, and 7 days after injection and skin near injection site and inguinal lymph nodes isolated, disaggregated and analyzed by flow cytometry. ( (A) Gating strategy for the in vivo adoptive-transfer analysis. Draining lymph node suspensions were gated sequentially on cells by FSC/SSC, singlets, live cells, lymphocytes, and CD11c⁺ cells. DiO fluorescence represents donor-cell membrane-associated signal, whereas DiD fluorescence represents microparticle-associated signal. DiO⁺, DiD⁺, and DiO⁺DiD⁺ events were quantified as donor-label-associated, MP-associated, and double-positive populations, respectively. (B) Representative flow plots for treatment groups on day 5 for CD11c+ lymphocytes (left) or skin (right). Breakdown of DiD and/or DiO+ cells from CD11c+ lymphocytes on (C-F) day 1, 3, 5 and 7. Breakdown of DiD and/or DiO+ live skin cells on (G-J) day 1, 3, 5 and 7. (K) PD-L1+ cell population of CD11c+ lymphocytes. L. CD11c+ population over time. Note significant increase for PPI MP group compared to Blank MP on day 7. M. % CD11c+ of MP+ lymphocytes. N= 4-5 mice per group (N) DC^PPI MP^, DC^Dex^ and untreated BMDCs on day 5 post differentiation were stained for CCR7 and MFI normalized to untreated BMDCs. N=3 Significance was determined by two-way ANOVA with and Sidak multiple comparisons for L and tukey test for multiple comparisons for N. ***p<10^-3^, ****p<10^-4^.

**Supplementary Figure 11.** Gating strategy for the analyses shown in Fig. 5A-H. Cells were first gated on lymphocytes by FSC/SSC, followed by singlets, live cells, and CD45⁺ leukocytes. From the live singlet CD45⁺ population, CD3⁺ T cells, CD19⁺ B cells, and CD11c⁺ DCs were identified in parallel. Within CD3⁺ T cells, CD4⁺ and CD8⁺ subsets were identified, and Tregs were defined as CD4⁺CD25⁺CD127loFoxP3⁺ cells. OVA323-339 MHC II tetramer staining was evaluated in CD4⁺ and Treg populations, whereas OVA257-264 MHC I tetramer staining was evaluated in CD8⁺ T cells. PD-1 and CTLA-4 expression was assessed in the indicated T-cell subsets. Representative FMO controls are shown for the indicated gates.

**Supplementary Figure 12.** PD-1 and CTLA-4 analysis of T cells from figure 5A-H. (A-B) Representative histogram overlays of PD-1 (A) and CTLA-4 (B) expression across total CD3⁺ T cells, CD4⁺ T cells, total Tregs, OVA323-339 tetramer⁺ CD4⁺ T cells, OVA323-339 tetramer⁺ Tregs, CD8⁺ T cells, and OVA257-264 tetramer⁺ CD8⁺ T cells from a representative DCPPI MP-treated mouse. (C)% PD-1+ or (D) CTLA-4+ T cells of all CD45+, CD3+ live lymphocytes. MFI of various T cell populations of (E) PD-1 or (F) CTLA-4. (G) % CD11c+ or (H) CD19+ cells of live singlet CD45+ cells.

**Supplementary Figure 13.** Supplemental T cell proliferation assay analysis. . (A) Flow cytometry gating strategy used to identify live singlet CD3⁺ T cells, CD4⁺ and CD8⁺ subsets, and proliferated cells based on Cell Proliferation Dye eFluor 670 dilution. Representative unstimulated and anti-CD3/CD28-stimulated controls are shown. (B) CD4⁺ cells and (C) CD8⁺ cells as percentages of CD3⁺ T cells. (D) Percentage of proliferated CD4⁺ T cells and (E) proliferated CD8⁺ T cells. (F) CD69 MFI on CD4⁺ T cells and (G) CD69 MFI on CD8⁺ T cells. Panels D/E and F/G provide the subset-level analyses corresponding to the total T-cell proliferation and CD69 measurements in Fig. 5J/K, respectively.

**Supplementary Tables**

**Supplementary Table 1.** EDS Analysis of Blank MP

**Supplementary Table 2.** EDS Analysis of PPI MP

**Supplementary Table 3.** Loading Analysis of Dex MP

| Compound | Encapsulation Efficiency (%) | Loading (wt. %) |
| --- | --- | --- |
| Dex | 8.32±0.65 | 14.1±1.1 |
| OVA | 8.8±0.47 | 3.3±0.35 |

**Supplementary Table 4. In vivo Treg flow cytometry panel.**

| **Marker** | **Clone** | **Product #** | **Fluorophore** | **Dilution** |
| --- | --- | --- | --- | --- |
| CD127 | A7R34 | 404-1271-82 | BV421 | 1:100 |
| CD4 | RM4-5 | 414-0042-80 | BV480 | 1:1000 |
| CD25 | PC61.5 | 63-0251-82 | BV605 / SB600 | 1:200 |
| PD-1 | J43 | 416-9985-80 | BV650 | 1:100 |
| CD45 | 30-F11 | 407-0451-80 | BV711 | 1:1000 |
| CD3 | 17A2 | 78-0032-82 | BV786 / SB780 | 1:500 |
| CD8 (CD8α) | 53-6.7 | 11-0081-81 | FITC | 1:500 |
| MHC II tetramer | N/A | NIH Tetramer Core Facility (Custom) | PE | 1:500 |
| CTLA-4 | UC10-4B9 | 61-1522-82 | PE–eFluor 610 | 1:200 |
| FoxP3 | FJK-16s | 15-5773-80 | PE-Cy5 | 1:200 |
| CD11c | N418 | 25-0114-81 | PE-Cy7 | 1:500 |
| MHC I tetramer | N/A | NIH Tetramer Core Facility (Custom) | APC | 1:500 |
| CD19 | 1D3 (eBio1D3) | M004T02R04A | NovaFluor Red 710 | 1:300 |
| Live/Dead | N/A | L34975 | Fixable Near-IR | 1:1000 |

**Supplementary Table 5. Ex vivo proliferation flow cytometry panel.**

| **Marker** | **Clone** | **Product #** | **Fluorophore** | **Dilution** |
| --- | --- | --- | --- | --- |
| CD4 | RM4-5 | 53-0042-82 | Alexa Fluor 488 | 1:250 |
| CD8 (CD8α) | 53-6.7 | 12-0081-82 | PE | 1:1000 |
| CD3 | 17A2 | 15-0032-82 | PE-Cy5 | 1:500 |
| CD69 | H1.2F3 | 25-0691-82 | PE-Cy7 | 1:500 |
| Proliferation | N/A | 65-0840-85 | eFluor 670 | 1:500 |
| Live/Dead | N/A | L34975 | Fixable Near-IR | 1:1000 |
